# Serum microbiome-related metabolites—including short-chain fatty acids and indole derivatives—predict outcome and delayed cerebral ischemia after aneurysmal subarachnoid hemorrhage: a two-timepoint LC–MS study

**DOI:** 10.3389/fneur.2026.1768108

**Published:** 2026-04-07

**Authors:** Blanka Nagybanyai-Nagy, Roland Tengölics, Csilla Sajben, Dominika Olasz, Attila Schwarcz, Tihamer Molnar, Peter Csecsei

**Affiliations:** 1 Department of Anaesthesiology and Intensive Care, University of Pecs Medical School, Pécs, Hungary; 2 Metabolomics Laboratory, Core Facilities, HUN-REN Biological Research Centre Szeged, Szeged, Hungary; 3 Department of Neurosurgery, University of Pecs Medical School, Pécs, Hungary

**Keywords:** aneurysmal subarachnoid hemorrhage, delayed cerebral ischemia, indole-3-propionic acid, outcome, short-chain fatty acids, tryptophan

## Abstract

**Background:**

Delayed cerebral ischemia (DCI) remains a major determinant of poor outcome after aneurysmal subarachnoid hemorrhage (aSAH). Growing evidence suggests that gut microbiota–derived metabolites, including short-chain fatty acids (SCFAs) and tryptophan-related indole compounds, modulate neuroinflammation and cerebrovascular vulnerability. However, their temporal dynamics and clinical relevance after aSAH are insufficiently characterized.

**Methods:**

In this prospective observational study, 80 consecutive patients with aSAH were enrolled at a tertiary neurocritical care center. Serum concentrations of SCFAs (propionic, butyric, isobutyric, valeric, isovaleric, caproic acids) and tryptophan-derived metabolites (tryptophan, indole-3-propionic acid [IPA], indole-3-acetic acid, indole-3-lactic acid) were quantified using LC–MS on Day 1 and Day 9 after hemorrhage. Functional outcome at 3 months was assessed using the modified Rankin Scale (mRS), and DCI was diagnosed according to consensus criteria. Associations were analyzed using non-parametric statistics, ROC analyses, and multivariable logistic regression adjusted for established clinical confounders.

**Results:**

Patients with unfavorable 3-month outcomes (mRS 4–6) showed significantly lower Day 1 levels of propionic, isobutyric, and isovaleric acids, persistently reduced tryptophan at both time points, and markedly lower IPA concentrations on Day 9. DCI was associated with reduced tryptophan and propionic acid levels on both days and a pronounced decrease in IPA on Day 9. Tryptophan and propionic acid demonstrated excellent discriminative performance for outcome and DCI (AUCs up to 0.99). In multivariable models, low Day 1 propionic acid and low Day 9 IPA independently predicted unfavorable outcome, while Day 9 tryptophan, IPA, and propionic acid independently predicted DCI.

**Conclusion:**

Distinct temporal alterations in gut microbiota–derived metabolites after aSAH are strongly associated with functional outcome and DCI. SCFAs and tryptophan-related metabolites—particularly propionic acid, tryptophan, and IPA—emerge as promising biomarkers and potential mechanistic mediators in secondary brain injury after aSAH.

## 1 Introduction

Aneurysmal subarachnoid hemorrhage (aSAH) is a devastating cerebrovascular event, accounting for approximately 5–10% of all strokes yet contributing disproportionately to morbidity and mortality (1). Epidemiological analyses indicate a 28-day mortality approaching 25–30%, and among survivors, nearly half never regain their premorbid level of functioning (1, 2). Beyond the initial hemorrhage, secondary complications play a critical role in patient outcomes. Among these, delayed cerebral ischemia (DCI) remains the most clinically significant late sequela, substantially worsening neurological recovery and long-term prognosis (3). In recent years, increasing attention has been directed toward the gut–brain axis as a key modulator of neuroinflammatory and neurovascular processes relevant to acute brain injury (4, 5). The gut microbiota and its metabolites—including short-chain fatty acids (SCFAs; such as propionic, butyric, isobutyric, valeric, isovaleric, and caproic acids) and tryptophan-derived indole compounds (e.g., indole-3-propionic acid (IPA), indole-3-acetic acid (IAA), indole-3-lactic acid (ILA), and tryptophan itself)—have emerged as potential contributors to cerebrovascular vulnerability and post-stroke recovery (4–7). SCFAs are known to reinforce the intestinal barrier, exert anti-inflammatory effects, and modulate systemic immune responses, thereby influencing the severity and trajectory of neurological injury (4–8). Clinical and experimental studies in ischemic stroke have shown that reduced abundance of SCFA-producing bacteria and lower fecal SCFA levels correlate with more severe neurological deficits and poorer 90-day outcomes (7, 8). Conversely, transplantation of SCFA-rich microbiota in animal models enhances post-stroke neurobehavioral recovery, supporting a causal role for microbiota-derived metabolites in neuroprotection (9). Disturbances in gut microbiota–mediated tryptophan metabolism play a significant role in ischemic stroke pathology through effects on the gut–brain axis, and targeting key tryptophan metabolic pathways and their metabolites may offer promising therapeutic avenues (10), as indole-3-propionic acid (IPA) emerges as a particularly promising neuroprotective tryptophan metabolite that acts through antioxidant, anti-inflammatory, receptor-mediated, and neurotrophic mechanisms to disrupt the gut–inflammation–brain cycle and potentially delay or mitigate neurodegenerative and ischemic brain disorders (11). Klepinowski et al. demonstrated that patients who developed cerebral vasospasm or delayed cerebral ischemia after aSAH displayed early alterations in gut microbiome diversity—particularly reduced butyrate-producing taxa—suggesting a microbial signature associated with these complications (12). A recent study found that patients with ruptured intracranial aneurysms exhibit significantly different gut microbial compositions compared with those with unruptured aneurysms, including shifts in SCFA-producing bacterial taxa and associated metabolic pathways (13). Taken together, these observations highlight a biologically plausible and clinically relevant interplay between gut microbiota, its metabolic products, and cerebrovascular disease processes. In this context, our study aimed to quantify serum levels of key SCFAs and tryptophan-derived metabolites (IPA, tryptophan, indole-acetic acid, indole-lactic acid, propionic acid, butyric acid, isobutyric acid, valeric acid, isovaleric acid, and caproic acid) at two time points following aSAH (Day 1 and Day 9), using liquid chromatography–mass spectrometry (LC–MS), an analytical platform combining chromatographic separation with mass spectrometric detection for sensitive and specific metabolite quantification. By characterizing their temporal profiles and associations with clinical outcomes, we sought to explore their potential roles as biomarkers or mechanistic mediators in the pathophysiology of aSAH and DCI. Based on prior evidence linking gut microbiota–derived metabolites to neuroinflammation, vascular dysfunction, and post-stroke recovery, we formulated the *a priori* hypothesis that reduced circulating levels of SCFAs and microbiota-derived tryptophan metabolites—particularly propionic acid, tryptophan, and IPA—would be associated with unfavorable 3-month functional outcome after aSAH. Furthermore, we hypothesized that early alterations in these metabolites would predict the subsequent development of DCI, reflecting a dysregulated gut–brain–immune axis contributing to secondary brain injury.

## 2 Materials and methods

### 2.1 Participants and study design

Institutional ethical approval for this prospective observational study was obtained prior to patient enrollment (IV/8468-1/2021/EKU, 27.10.2021 and BM/4629-1/2024), and written informed consent was secured from all patients or their legal representatives before participation. Consecutive adults diagnosed with aSAH at our tertiary neurocritical care center were enrolled between September 2023 and July 2025. Inclusion criteria were: (i) age ≥18 years; (ii) confirmation of aSAH by non-contrast head CT and verification of an intracranial aneurysm by CTA or DSA; and (iii) diagnosis established within 24 h following ictus. Patients with traumatic SAH, pregnancy, delayed hospital admission (>24 h after ictus), untreated aneurysms, arteriovenous malformation–related bleeding, absence of informed consent, or underlying chronic systemic diseases—including malignancy, hepatic or renal insufficiency, chronic pulmonary disease, inflammatory bowel disease, or any known chronic gastrointestinal disorder—were excluded. Additional exclusion criteria included chronic or acute infection at admission, as well as rerupture or clinical deterioration after the initial bleeding event. For all eligible patients, comprehensive clinical data were systematically collected, including demographic characteristics, vascular risk factors, presenting symptoms, neurological severity scores (WFNS and modified Fisher scale), radiological findings, and all neurocritical care interventions performed during hospitalization such as mechanical ventilation, extraventricular or lumbar drainage, and decompressive surgery. Laboratory parameters at admission—including C-reactive protein, creatinine, white blood cell count, and neutrophil–lymphocyte ratio—were also recorded. Blood samples for metabolomic measurements were drawn at two prespecified time points: Day 1 (D1), within the acute phase of hemorrhage (arterial blood sampling was performed 24 h after the ictus), and Day 9 (D9), corresponding to the period of highest DCI risk (arterial blood sampling was performed within 216 ± 4 h after the ictus). Serum levels of short-chain fatty acids (SCFAs) and tryptophan-derived indole metabolites were quantified using LC–MS analysis. All patients were followed for 3 months after the index event using structured telephone or in-person evaluations, conducted by trained assessors who had completed formal mRS certification and were blinded to biomarker data. No formal *a priori* sample size calculation was performed. This study was designed as a prospective exploratory observational investigation, and patient enrollment was based on consecutive inclusion during the predefined recruitment period at our tertiary neurocritical care center. At the time of study planning, insufficient prior data were available on circulating SCFA and indole metabolite effect sizes in aSAH to permit reliable power estimation. The sample size therefore reflects the total number of eligible patients enrolled within the study period.

### 2.2 Clinical definitions, data collection methods and outcome evaluation

Clinical data were collected prospectively from all enrolled patients at admission and throughout the acute hospitalization period. Demographic variables, vascular risk factors (hypertension, diabetes mellitus, ischemic heart disease, smoking history), and clinical presentation features—including loss of consciousness at ictus—were systematically recorded. Initial neurological severity was assessed using the World Federation of Neurosurgical Societies (WFNS) scale, while the extent of subarachnoid blood was graded using the modified Fisher (mFisher) scale. Aneurysm location, radiological findings, and the need for neurosurgical or neurocritical care interventions—such as lumbar drainage, extraventricular drainage (EVD), mechanical ventilation, and decompressive craniotomy—were extracted from clinical charts and imaging reports. Laboratory parameters obtained at admission included C-reactive protein, creatinine, white blood cell count, and neutrophil–lymphocyte ratio. The primary clinical outcome was functional status at 3 months, assessed using the modified Rankin Scale (mRS), with favorable outcome defined as mRS 0–3 and unfavorable outcome as mRS 4–6. Delayed cerebral ischemia (DCI) was defined according to the 2010 consensus criteria proposed by Vergouwen et al. (3) as either: (i) the occurrence of a new focal neurological deficit or a decrease of at least 2 points on the Glasgow Coma Scale lasting for ≥1 h, not attributable to rebleeding, hydrocephalus, seizures, metabolic disturbances, infection, or other identifiable causes after clinical and radiological evaluation; and/or (ii) the presence of a new cerebral infarction on follow-up CT or MRI not present on the immediate post-treatment scan and not attributable to procedural complications or other causes. Symptomatic angiographic vasospasm in the absence of clinical deterioration or radiologically confirmed infarction was not considered sufficient for DCI diagnosis. The diagnosis was established by agreement of at least two experienced neurointensivists blinded to biomarker data. These outcome categories were used for all subsequent comparative analyses of metabolite concentrations. The occurrence of infectious complications during hospitalization was also documented. Blood samples for metabolomic analyses were obtained on Day 1 (D1) and Day 9 (D9) following aSAH. Serum was processed and stored according to standardized protocols prior to LC–MS analysis. Quantified metabolites included short-chain fatty acids (propionic, butyric, isobutyric, valeric, isovaleric, and caproic acids) and indole-derived tryptophan metabolites (indole-3-propionic acid, tryptophan, indole-3-acetic acid, and indole-3-lactic acid).

### 2.3 LC–MS-based metabolite determination

Liquid chromatography–mass spectrometry (LC–MS) is an analytical technique that couples chromatographic separation of compounds with mass-based detection, allowing precise quantification of small-molecule metabolites in complex biological samples. Short-chain fatty acids (SCFAs) and indole derivatives were quantified following an established method (14) with several modifications. Human serum samples were thawed at 4 °C under constant shaking using a Genie 2 Vortex (Scientific Industries, Bohemia, NY, United States) for 30 min. Sixty microliters of serum were mixed with 420 μL ice-cold methanol (Supelco 1.06035) in a 1.5 mL microcentrifuge tube for protein precipitation. Samples were vortexed at 1000 rpm for 10 min, then centrifuged at 15,000 rpm for 10 min at 4 °C to obtain the serum extract. Sixty microliters of the resulting supernatant were combined on ice with 30 μL of 0.2 M 3-NPH and 30 μL of 0.12 M EDC. The reaction was incubated at 40 °C for 25 min without shaking, followed by centrifugation at 15,000 rpm for 10 min at 4 °C. The clarified supernatant was transferred into HPLC vials (VP91, Zhejiang Aijiren Technology Inc., Qujiang, Quzhou, Zhejiang, China) and sealed with PTFE/silicone septa (SC9291, Zhejiang Aijiren Technology Inc.). SCFAs were separated on a Waters HSS T3 column (1.7 μm, 2.1 × 100 mm) using a Waters ACQUITY Premier UPLC coupled to a Waters TQS-Micro tandem mass spectrometer. Mobile phase A consisted of LC–MS–grade water (VWR 83645.320) with 0.1% formic acid (Sigma 5.33002), and mobile phase B consisted of 0.1% formic acid in 2-propanol (VWR 84881.320):acetonitrile (VWR 83640.320). The injection volume was 3 μL and the flow rate was 0.3 mL/min. The LC gradient was programmed as follows: 0–0.2 min, 0% B; 0.2–0.7 min, 0–15% B; 0.7–2 min, 15% B; 2–6 min, 15–30% B; 6–12 min, 30–72% B; 12–12.4 min, 72–100% B; 12.4–14 min, 100% B; 14–14.1 min, 100–0% B; 14.1–15.6 min, 0% B. The autosampler was maintained at 10 °C and the column at 55 °C. Mass spectrometric detection was performed in negative electrospray ionization mode. Source settings were: capillary voltage 0.5 kV, desolvation temperature 600 °C, desolvation gas flow 1,000 L/h, and cone gas flow 1 L/h. Data were acquired in multiple-reaction monitoring mode using previously described transitions (14). The cone voltage was set to 10 V for all target analytes. Collision energy was set to 10 V for propionic acid and 20 V for all other SCFAs. Calibration was performed using an external dilution series of an SCFA mix as described (14). Samples were analyzed in four experimental batches. Each batch included three pooled QC samples and one blank to monitor and correct for batch effects. Quantitative data were obtained using an eight-point external calibration curve fitted either with linear regression or a second-order polynomial model. All R2 values were above 0.99. For LOQ estimation, we evaluated the signal-to-noise (S/N) ratios of analyte peaks across calibration levels. In parallel, we assessed S/N values in blank samples. LOQ was defined as the lowest calibration level with a signal-to-noise ratio ≥10 and at least twofold higher than the blank signal. Quantitative data were normalized using EigenMS batch normalization in MetaboAnalyst 6.0 (15). Acetic acid was excluded from downstream analysis due to high blank signal. Analytical performance of the transferred LC–MS method was evaluated by assessing calibration linearity, signal-to-noise ratios, and technical reproducibility using pooled QC samples across analytical batches. Calibration curves demonstrated excellent linearity (*R*^2^ > 0.99), and limits of quantification were defined based on signal-to-noise criteria. Technical reproducibility assessed from repeated QC injections showed low analytical variability. Statistical analysis and data visualization were performed using IBM SPSS Statistics v25 (IBM, Armonk, New York, United States) and GraphPad Prism 10 (GraphPad Software, LLC, San Diego, California, United States).

### 2.4 Statistical analysis

All statistical analyses were conducted using IBM SPSS Statistics version 25 (IBM Corp., Armonk, NY, United States) and GraphPad Prism version 10 (GraphPad Software, LLC, San Diego, CA, United States). Continuous variables were inspected for distributional characteristics using histograms, Q–Q plots, and the Shapiro–Wilk test. Because most biochemical and clinical parameters exhibited non-normal distributions, non-parametric tests were applied throughout the analyses unless otherwise indicated. Categorical variables were analyzed using contingency-table statistics. A two-sided *p*-value <0.05 was considered statistically significant. Baseline demographic and clinical characteristics were summarized as mean ± standard deviation (SD) for normally distributed variables, median with interquartile range (IQR) for non-normally distributed data, and frequencies with percentages for categorical variables. Between-group comparisons (favorable vs. unfavorable 3-month functional outcome) were performed using: Independent-samples *t*-tests for continuous variables with approximate normality, Mann–Whitney U tests for non-normally distributed continuous data (e.g., WFNS, mFisher score, inflammatory markers), Chi-square tests or Fisher’s exact tests for categorical variables (e.g., hypertension, diabetes, aneurysm location, DCI), as appropriate. Serum concentrations of SCFAs and indole-derived metabolites measured on Day 1 and Day 9 were compared between clinical subgroups. For each metabolite, differences between groups (favorable vs. unfavorable outcome; DCI vs. No-DCI) were analyzed using Mann–Whitney U tests due to non-parametric distributions and unequal variances. Results are illustrated as box-and-whisker plots with median, IQR, and full data range. Statistical significance is indicated using the standard notation (*p* < 0.05; *p* < 0.01; *p* < 0.001; *p* < 0.0001). Given the number of biomarker comparisons, false discovery rate (FDR) correction using the Benjamini–Hochberg procedure was applied as a sensitivity analysis. To assess the discriminative performance of individual metabolites for predicting (i) unfavorable 3-month outcome and (ii) the development of delayed cerebral ischemia (DCI), ROC curve analyses were conducted for each metabolite at both time points (Day 1 and Day 9). For each ROC model, the following were computed: Area under the curve (AUC) with 95% confidence intervals (DeLong method), Sensitivity and specificity at optimal cut-off values defined by Youden’s index, Corresponding *p*-values for AUC significance testing. ROC analyses were performed in a univariable manner for each metabolite separately and were not adjusted for clinical covariates. Adjustment for established clinical predictors was conducted exclusively in the multivariable logistic regression models. To evaluate the independent association between selected metabolites and clinical outcomes, multivariable logistic regression models were constructed for: 3-month functional outcome (favorable vs. unfavorable), occurrence of DCI. Each model included clinical covariates chosen *a priori* based on known prognostic relevance and to control for confounding: Outcome prediction models (Table 1): age, WFNS grade, and infection status. DCI prediction models (Table 2): age, hypertension, and modified Fisher score. For each metabolite (analyzed in separate models): Odds ratios (ORs) and 95% confidence intervals (CIs) were calculated per unit increase in metabolite concentration, Wald χ^2^ statistics assessed predictor significance. Model performance was summarized by Nagelkerke’s *R*^2^, reflecting explained variance. To evaluate the interrelations among SCFAs and indole-derived metabolites at both time points, Spearman’s rank correlation coefficients were calculated. Correlation matrices were visualized as heatmaps, using a color gradient from −1 to +1. Autocorrelations between Day 1 and Day 9 levels were examined to assess temporal stability of metabolite patterns.

**Table 1:** SCFA levels, IPA and 3-month outcomes in aSAH patients: ORs at D1 and D9.

Variable	OR (95% CI)	Wald χ^2^	*P*-value	Model fit (Nagelkerke *R*^2^)
Propionic acid D1	0.49 (0.29–0.81)	7.6	0.006	0.68
IPA D9	0.09 (0.02–0.38)	10.7	0.001	0.70
Tryptophan D1	0.53 (0.28–1.00)	3.8	0.051	0.64
Isobutyric acid D1	0.95 (0.62–1.48)	0.04	0.842	0.64
Isobutyric acid D9	0.67 (0.49–0.90)	6.6	0.01	0.67
Isovaleric acid D1	0.28 (0.1–0.85)	4.9	0.026	0.66

ORs (odds ratios, 95% CI: confidence intervals), Wald χ^2^, and *p*-values for SCFAs (short-chain fatty acids: propionic acid, IPA: indole-3-propionic acid, isobutyric acid, isovalerianic acid) measured at D1 (day 1) and D9 (day 9) in aSAH patients; model fit indicated by Nagelkerke *R*^2^. IPA, indol-3-propionic acid; aSAH, aneurysmal subarachnoid hemorrhage; OR, odds-ratio.

**Table 2:** Binary logistic regression analysis of metabolite levels associated with the development of DCI in aSAH patients (adjusted for age, hypertension, and mFisher score).

Variable	OR (95% CI)	Wald χ^2^	*P*-value	Model fit (Nagelkerke *R*^2^)
IPA D1	0.55 (0.17–1.82)	0.9	0.329	0.34
IPA D9	0.13 (0.02–0.86)	4.5	0.034	0.5
Trytophane D1	0.87 (0.38–1.9)	0.1	0.731	0.32
Tryptophan D9	0.02 (0.01–0.05)	6.7	<0.001	0.43
Propionic acid D1	0.66 (0.41–1.09)	2.6	0.108	0.37
Propionic acid D9	0.51 (0.32–0.81)	8.2	0.004	0.52
Isovaleric acid D1	0.31 (0.1–0.8)	3.9	0.048	0.41
Caproic acid D1	0.24 (0.08–0.77)	5.7	0.017	0.46

ORs (odds ratios, 95% CI: confidence intervals), Wald χ^2^, and *p*-values for SCFAs (short-chain fatty acids: propionic acid, IPA: indole-3-propionic acid, isobutyric acid, isovalerianic acid) measured at D1 (day 1) and D9 (day 9) in aSAH patients; model fit indicated by Nagelkerke *R*^2^. IPA, indol-3-propionic acid; aSAH, aneurysmal subarachnoid hemorrhage; OR, odds-ratio.

## 3 Results

### 3.1 Patients characteristics

A total of 131 patients were screened for eligibility. Of these, patients were excluded for the following reasons: traumatic subarachnoid hemorrhage (*n* = 4), delayed hospital admission (*n* = 6), arteriovenous malformation–related hemorrhage (*n* = 1), chronic systemic disease (*n* = 12), chronic obstructive pulmonary disease (*n* = 3), chronic gastrointestinal diseases (including inflammatory bowel disease, celiac-related disorders, and gastritis; *n* = 4), incomplete biomarker sampling (*n* = 11), loss to follow-up (*n* = 6), aneurysm rerupture prior to treatment (*n* = 1), and chronic infection or undefined immunological disease (including pyelonephritis; *n* = 3). After exclusions, 80 patients were included in the final analysis. All included patients underwent endovascular treatment; no patients were treated with open surgical clipping. The mean age of the total cohort was 58 ± 11 years, with patients in the favorable outcome group being slightly younger (56 ± 9 years) than those in the unfavorable group (60 ± 13 years). Females represented 74% of the study population, accounting for 64% of the favorable group and 84% of the unfavorable group. Hypertension was present in 56% of all patients, while diabetes occurred in 11%, the latter being more frequent among those with unfavorable outcomes (18% vs. 5%). Smoking was reported in 49% of patients, with comparable proportions across outcome groups. Ischemic heart disease was documented in 50% of the cohort, occurring in 43% of favorable and 57% of unfavorable cases. Loss of consciousness during ictus was observed in 35% of all patients. Baseline clinical severity differed markedly between groups: the median WFNS score was 1 (IQR 1–2) in the favorable group and 4 (IQR 2–5) in the unfavorable group, while the median modified Fisher score was 2 (IQR 2–3) and 3 (IQR 3–4), respectively. Inflammatory markers showed notable differences, with median C-reactive protein levels of 7 mg/L (IQR 2–13) in the favorable group and 29 mg/L (IQR 9–73) in the unfavorable group, and neutrophil–lymphocyte ratio values of 4.2 (IQR 3–7) versus 9.1 (IQR 5–12). Among infected patients treated with antibiotics (*n* = 31), antibiotic therapy was initiated a median of 7 days after D1 sampling (IQR 7–8; range 4–10). The need for extraventricular drainage (10% vs. 71%) and mechanical ventilation (7% vs. 82%) was substantially more common in the unfavorable outcome group, and delayed cerebral ischemia and infections were also more prevalent in this group (53 and 68%, respectively) (Table 1).

### 3.2 Associations of SCFAs, IPA, and tryptophan metabolites with 3-month outcome after aSAH

**Figure 1:**
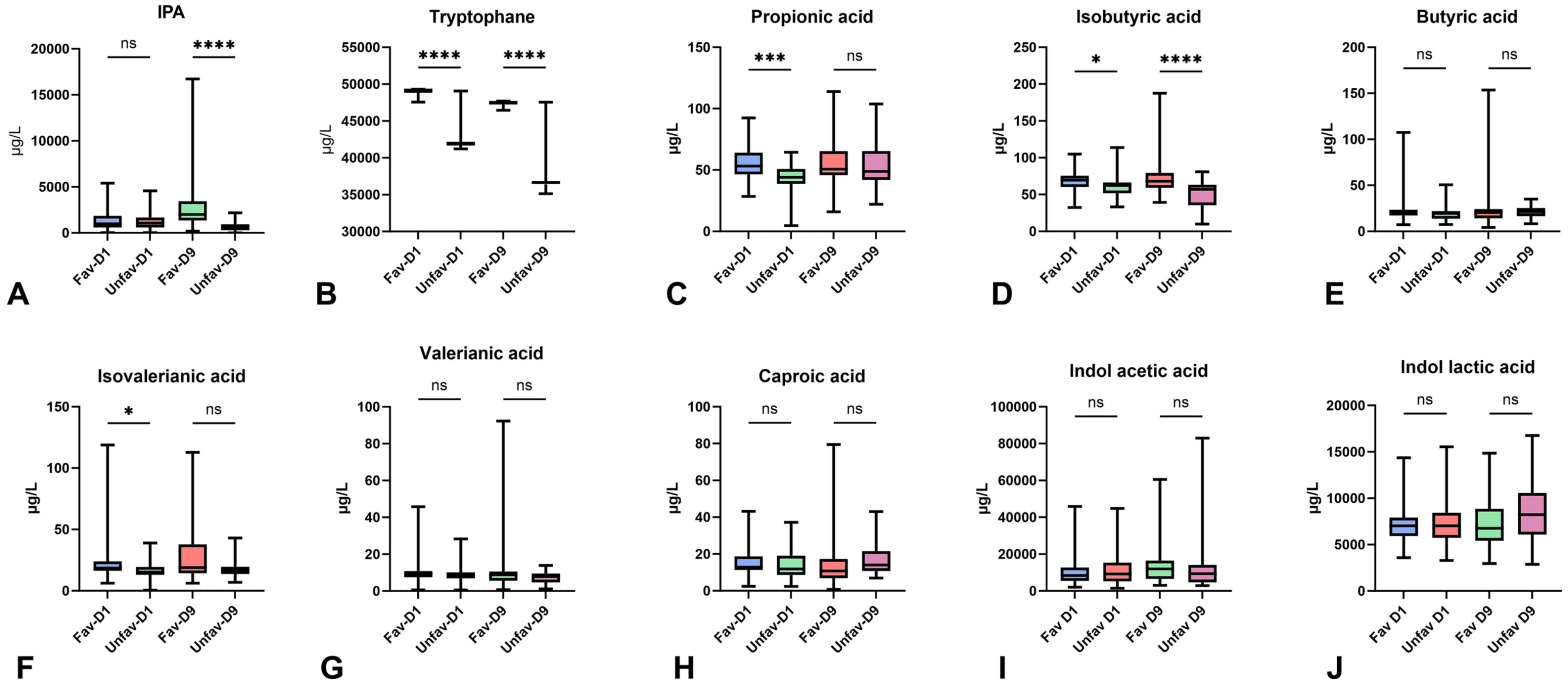
IPA, tryptophan, and short-chain fatty acid concentrations on Day 1 and Day 9 according to 3-month functional outcome in patients with aSAH. Boxplots illustrating serum concentrations of IPA, tryptophan, and short-chain fatty acids (SCFAs) in aSAH patients with favorable (Fav) and unfavorable (Unfav) 3-month outcomes, measured on Day 1 (D1) and Day 9 (D9). Lines above the boxplots indicate between-group comparisons for each time point. Statistical significance is denoted as follows: **** *p* < 0.0001; *** *p* < 0.001; ** *p* < 0.01; * *p* < 0.05; ns, not significant; IPA, indole-3-propionic acid. **(A)** Indole-3-propionic acid (IPA); **(B)** Tryptophan; **(C)** Propionic acid; **(D)** Isobutyric acid; **(E)** Butyric acid; **(F)** Isovalerianic acid; **(G)** Valeric acid; **(H)** Caproic acid; **(I)** Indole-3-acetic acid; **(J)** Indole-3-lactic acid. In the favorable outcome group (Fav), the number of patients was *n* = 42, while in the unfavorable outcome group (Unfav), *n* = 38.

Figure 1 presents the serum concentrations of short-chain fatty acids (SCFAs), IPA and tryptophan on D1 and D9 according to 3-month functional outcome. For IPA (Figure 1A), no significant difference appeared between Fav-D1 and Unfav-D1 (ns), whereas Fav-D9 showed substantially higher concentrations than Unfav-D9 (*p* < 0.0001). Tryptophan (Figure 1B) levels were markedly higher in the Fav-D1 group compared with Unfav-D1 (*p* < 0.0001), and a similarly strong difference was observed between Fav-D9 and Unfav-D9 (p < 0.0001). Propionic acid (Figure 1C) levels were significantly lower in the unfavorable outcome group on Day 1 (*p* < 0.001), whereas no difference was observed on D9. Isobutyric acid (Figure 1D) also differed between groups, with lower concentrations in the unfavorable group on D1 (*p* < 0.05) and on D9 (*p* < 0.0001). In contrast, butyric acid (Figure 1E) showed no significant difference at either time point. Isovaleric acid (Figure 1F) was significantly reduced in the unfavorable outcome group on D1 (*p* < 0.05), while Day 9 levels did not differ between groups. Valeric acid (Figure 1G) and caproic acid (Figure 1H) showed no significant differences on either Day 1 or Day 9 (all *p* > 0.05). Indole acetic acid (Figure 1I) levels did not differ significantly between groups at either time point (both comparisons marked as ns). Likewise, indole lactic acid (Figure 1J) displayed no significant differences between Fav and Unfav groups on day 1 or day 9 (ns for both comparisons).

**Figure 2:**
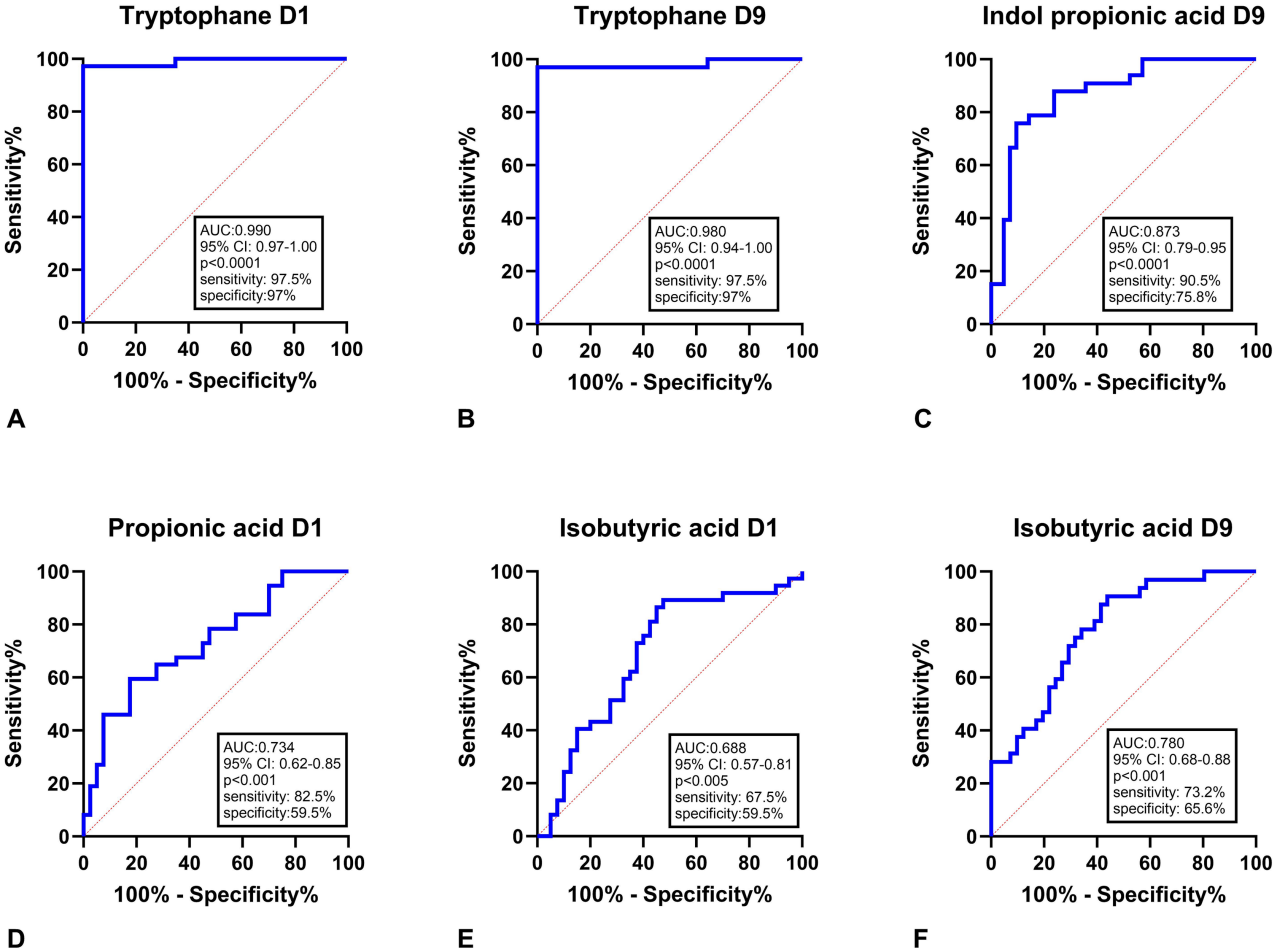
ROC curve analysis of tryptophan, indole- and short-chain fatty acid–related metabolites on Day 1 and Day 9 after aSAH in relation to the 3-month outcome. Each ROC panel includes the corresponding 95% confidence interval (CI), *p*-value, sensitivity, and specificity. **(A)** Tryptophan D1; **(B)** Tryptophan D9; **(C)** Indole propionic acid D9; **(D)** Propionic acid D1; **(E)** Isobutyric acid D1; **(F)** Isobutyric acid D9. AUC, area under the curve; CI, confidence interval; D1, Day 1; D9, Day 9.

The ROC analyses show that tryptophan (Figures 2A,B) provides excellent discriminatory performance at both time points. On day 1, tryptophan reached an AUC of 0.990 (95% CI: 0.97–1.00, *p* < 0.0001) with a sensitivity of 97.5% and specificity of 97%. Similarly, on day 9, its AUC remained high at 0.980 (95% CI: 0.94–1.00, *p* < 0.0001), again with 97.5% sensitivity and 97% specificity. Indole-3-propionic acid (Figure 2C) measured on day 9 showed an AUC of 0.873 (95% CI: 0.79–0.95, *p* < 0.0001), accompanied by 90.5% sensitivity and 75.8% specificity. Propionic acid (Figure 2D) on day 1 exhibited a moderate AUC of 0.734 (95% CI: 0.62–0.85, *p* < 0.001) with sensitivity and specificity of 82.5 and 59.5%, respectively. Isobutyric acid (Figures 2E,F) on day 1 showed an AUC of 0.688 (95% CI: 0.57–0.81, *p* < 0.005) with sensitivity of 67.5% and specificity of 59.5%, while on day 9 its AUC increased to 0.780 (95% CI: 0.68–0.88, *p* < 0.001) with sensitivity of 73.2% and specificity of 65.6% (Figure 2).

The multivariable logistic regression model—including age, WFNS grade, and the presence of infection as covariates—evaluated the association between selected metabolites and 3-month outcomes in patients with aSAH. Propionic acid on day 1 was significantly associated with 3-month outcome (OR = 0.49, 95% CI 0.29–0.81; Wald χ^2^ = 7.6; *p* = 0.006; *R*^2^ = 0.68). IPA measured on day 9 showed a strong association with the outcome (OR = 0.09, 95% CI 0.02–0.38; Wald χ^2^ = 10.7; *p* = 0.001; *R*^2^ = 0.70). Tryptophan on day 1 demonstrated a near-significant relationship (OR = 0.53, 95% CI 0.28–1.00; Wald χ^2^ = 3.8; *p* = 0.051; *R*^2^ = 0.64). Additional metabolites—such as isobutyric acid on day 1 (OR = 0.95, *p* = 0.842) and day 9 (OR = 0.67, *p* = 0.01), as well as isovaleric acid on day 1 (OR = 0.28, *p* = 0.026) showed variable associations with 3-month outcome, with Nagelkerke *R*^2^ values ranging from 0.64 to 0.67 (Table 2).

In incremental value analyses for 3-month outcome, adding Day 1 propionic acid to the baseline clinical model (including WFNS, age, and covariates) significantly improved model fit (Δχ^2^ = 13.45, df = 1, *p* < 0.001). Discrimination increased from an AUC of 0.862 (95% CI 0.770–0.954) for the clinical model alone to 0.916 (95% CI 0.845–0.986) after inclusion of propionic acid. Model calibration remained acceptable (Hosmer–Lemeshow *p* = 0.142). These findings indicate that Day 1 propionic acid provides incremental prognostic value beyond established clinical severity measures.

After false discovery rate (FDR) correction using the Benjamini–Hochberg procedure, the principal associations (Day 1 propionic acid, Day 1 and Day 9 tryptophan, and Day 9 IPA) remained statistically significant.

### 3.3 Associations of SCFAs, IPA, and tryptophan metabolites with DCI after aSAH

**Figure 3:**
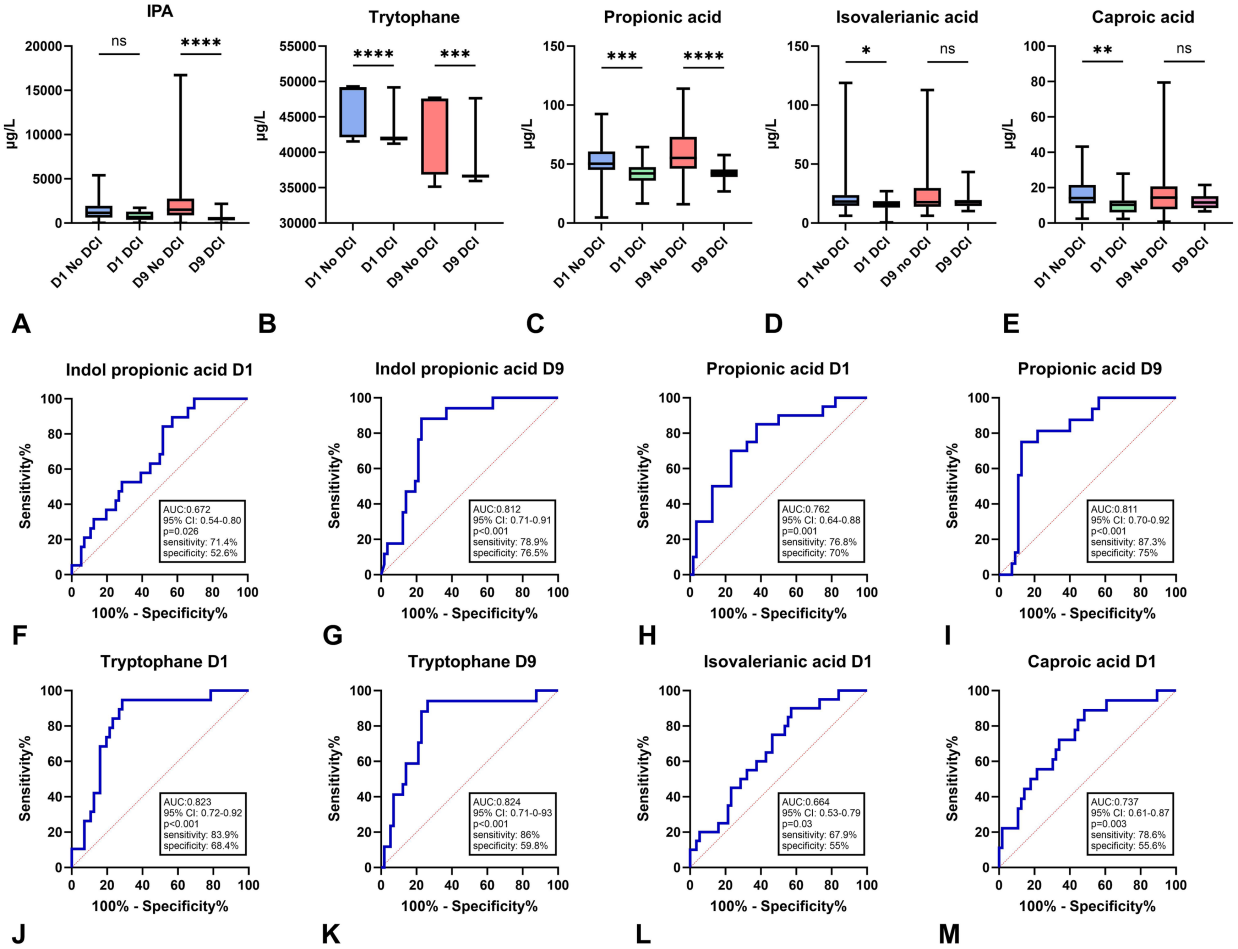
Metabolite profiles and their predictive performance in aSAH according to DCI status. Indol-3-propionic acid **(A)** serum concentrations on D1 and D9 comparing DCI and no-DCI groups, showing no difference on D1 and a highly significant increase in DCI patients on D9. Tryptophan **(B)** levels at D1 and D9, demonstrating strong and persistent group differences with higher values in the DCI group. Propionic acid **(C)** concentrations on D1 and D9, both time points showing marked elevations in DCI patients. Isovaleric acid **(D)** levels, with a significant group difference on D1 but no separation on D9. Caproic acid **(E)** concentrations, significantly differing between groups on D1 but not on D9. ROC curves for the examined metabolites: indole-propionic acid **(F,G)** on D1 (AUC = 0.672) and D9 (AUC = 0.812), propionic acid **(H,I)** on D1 (AUC = 0.762) and D9 (AUC = 0.811), tryptophan **(J,K)** on D1 (AUC = 0.823) and D9 (AUC = 0.824), isovaleric acid **(L)** on D1 (AUC = 0.664), and capric acid **(M)** on D1 (AUC = 0.737), demonstrating varying degrees of predictive performance. DCI, delayed cerebral ischemia; IPA, indole-3-propionic acid; D1/D9, Day 1/Day 9; AUC, area under the curve. In the DCI group, the number of patients was *n* = 22, while in the no-DCI group, *n* = 68.

The metabolite analyses revealed distinct concentration patterns between patients who developed DCI and those who did not (Figure 3). IPA levels (Figure 3A) showed no significant difference on D1 (ns), whereas a marked decrease was observed in DCI patients on D9 (*p* < 0.0001); correspondingly, IPA exhibited a moderate predictive performance (Figures 3F,G) on D1 (AUC = 0.672) and a substantially higher accuracy on D9 (AUC = 0.812). Tryptophan concentrations (Figure 3B) were significantly lower in DCI patients on both D1 (*p* < 0.0001) and D9 (*p* < 0.001), and its ROC curves (Figures 3J,K) demonstrated excellent discriminative ability at both time points (AUC = 0.823 on D1 and AUC = 0.824 on D9). Propionic acid (Figure 3C) similarly showed strong group differences, with elevated levels in patients without DCI on D1 (*p* < 0.001) and D9 (*p* < 0.0001); its predictive performance (Figures 3H,I) was consistently high across time points (AUC = 0.762 on D1 and AUC = 0.811 on D9). In contrast, isovaleric acid (Figure 3D) displayed a significant difference only on D1 (*p* < 0.05) but not on D9 (ns), while its predictive capacity (Figure 3L) remained moderate (AUC = 0.664 on D1). Caproic acid (Figure 3E) followed a similar pattern, showing a significant decrease in DCI patients on D1 (*p* < 0.01) but no difference on D9, with a moderate AUC of 0.737 on D1 (Figure 3M). Tryptophan and propionic acid emerged as the strongest discriminators between DCI and No-DCI patients, supported by both their consistent group differences and their high AUC values, while IPA exhibited improved predictive value at the later time point.

Similarly, for DCI, the key associations (Day 1 and Day 9 tryptophan, Day 9 propionic acid, and Day 9 IPA) remained significant after FDR correction.

An other multivariable logistic regression models—including age, hypertension, and mFisher score as covariates—evaluated the associations between several metabolites and the development of delayed cerebral ischemia (DCI) in patients with aSAH. IPA measured on day 1 showed no significant relationship with DCI (OR = 0.55, 95% CI 0.17–1.82; Wald χ^2^ = 0.9; *p* = 0.329; *R*^2^ = 0.34), whereas IPA on day 9 was significantly associated with DCI (OR = 0.13, 95% CI 0.02–0.86; Wald χ^2^ = 4.5; *p* = 0.034; *R*^2^ = 0.50). Tryptophan levels on day 1 were not related to DCI (OR = 0.87, *p* = 0.731), while day 9 tryptophan levels showed a strong association (OR = 0.02, 95% CI 0.01–0.05; Wald χ^2^ = 6.7; *p* < 0.001; *R*^2^ = 0.43). Propionic acid on day 1 did not reach statistical significance (OR = 0.66, *p* = 0.108), in contrast to its day 9 level, which was significantly associated with DCI (OR = 0.51, 95% CI 0.32–0.81; Wald χ^2^ = 8.2; *p* = 0.004; *R*^2^ = 0.52). In addition, isovaleric acid on day 1 (OR = 0.31, *p* = 0.048; *R*^2^ = 0.41) and caproic acid on day 1 (OR = 0.24, *p* = 0.017) were also significantly associated with DCI occurrence (Table 3). In incremental value analyses for DCI prediction, addition of tryptophan (Day 9) to the baseline clinical model significantly improved model fit (Δχ^2^ = 18.68, df = 1, *p* < 0.001). Discrimination increased from an AUC of 0.783 (95% CI 0.645–0.921) for the clinical model alone to 0.909 (95% CI 0.830–0.989) after inclusion of the biomarker. Model calibration remained acceptable (Hosmer–Lemeshow *p* = 0.645). These findings indicate substantial incremental prognostic value beyond established clinical predictors. Among the 22 patients who developed DCI, the median time from ictus to DCI was 9 days (IQR 8–10; range 5–13). Seven patients developed DCI before Day 9 sampling, five on Day 9, and ten after Day 9.

**Table 3:** Patients characteristics.

Variable	Total (*n* = 80)	Favorable (*n* = 42)	Unfavorable (*n* = 38)	*P*-value
Age (mean±SD)	58 ± 11	56 ± 9	60 ± 13	0.097
Female, *N* (%)	59 (74)	27 (64)	32 (84)	0.074
Hypertension, *N* (%)	45 (56)	21 (50)	24 (63)	0.266
Diabetes, *N* (%)	9 (11)	2 (5)	7 (18)	0.078
Smoking, *N* (%)	39 (49)	22 (52)	17 (45)	0.512
IHD, *N* (%)	40 (50)	18 (43)	22 (57)	0.263
Loss of consc. During ictus, *N* (%)	28 (35)	12 (29)	16 (42)	0.245
WFNS, median (IQR)	2 (1–4)	1 (1–2)	4 (2–5)	<0.001
mFisher score, median (IQR)	3 (2–4)	2 (2–3)	3 (3–4)	<0.001
Aneurysm location, *N* (%)				
Internal carotid artery	3 (4)	1 (2)	2 (5)	
Middle cerebral artery	23 (29)	12 (29)	11 (29)	
Anterior communicans artery	21 (26)	11 (26)	10 (26)	
Posterior communicans artery	9 (11)	6 (14)	3 (8)	
Anterior cerebral artery	5 (6)	3 (7)	2 (5)	
Vertebrobasilar	19 (24)	9 (21)	10 (26)	
C-reactive protein^a^, mg/L, median (IQR)	10 (4–45)	7 (2–13)	29 (9–73)	0.001
Creatinine^a^, μmol/L, median (IQR)	61 (52–76)	58 (52–71)	65 (52–78)	0.363
White blood cell count^a^, G/L, median (IQR)	10.1 (8–13)	9.9 (8–12)	10.3 (9–13)	0.369
Neutrophile-lymphocyte ratio^a^, median (IQR)	5.7 (4–10)	4.2 (3–7)	9.1 (5–12)	0.001
SBP^a^, median (IQR)	150 (131–170)	149 (134–170)	150 (131–170)	0.909
DBP^a^, median (IQR)	84 (79–94)	85 (80–94)	81 (78–92)	0.403
Lumbal drain, *N* (%)	42 (53)	22 (52)	20 (53)	1.000
Extraventricular drainage, *N* (%)	31 (39)	4 (10)	27 (71)	<0.001
Decompressive craniotomy, *N* (%)	3 (4)	0 (0)	3 (8)	0.109
Mechanical ventillation, *N* (%)	34 (43)	3 (7)	31 (82)	<0.001
Delayed cerebral ischemia, *N* (%)	22 (28)	2 (5)	20 (53)	<0.001
Infection, *N* (%)	34 (43)	8 (19)	26 (68)	<0.001

WFNS, World Federation of Neurosurgical Societies score; IQR, interquartile range; *N*, number; SD, standard deviation; IHD, ischemic heart disease; SBP, systolic blood pressure; DBP, diastolic blood pressure. The favorable outcome group was defined as patients with a modified Rankin Scale (mRS) score of 0–3, whereas the unfavorable outcome group included those with an mRS score of 4–6.

a On admission.

### 3.4 Correlation structure of indole-derived metabolites and SCFAs in aSAH patients at Day 1 and Day 9

**Figure 4:**
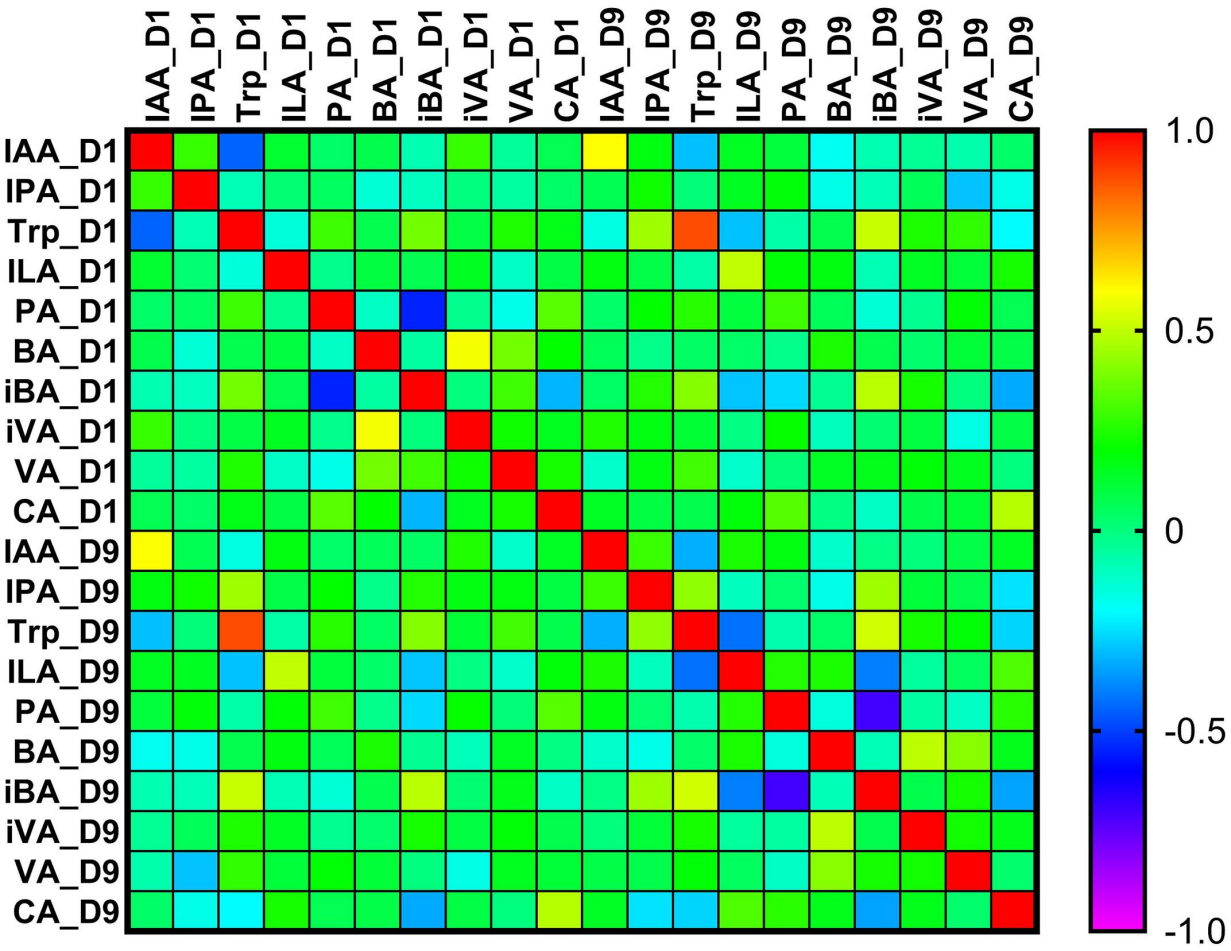
Correlation matrix of indole metabolites and short-chain fatty acids at D1 and D9 after aSAH. Indole-3-acetic acid (IAA), indole-3-propionic acid (IPA), tryptophan (Trp), indole-3-lactic acid (ILA), propionic acid (PA), butyric acid (BA), isobutyric acid (iBA), isovaleric acid (iVA), valeric acid (VA), caproic acid (CA). The color gradient ranges from −1.0 (strong negative correlation) to +1.0 (strong positive correlation), reflecting the degree of monotonic association captured by Spearman’s rho.

Heatmap depicts the correlation structure of indole-derived metabolites and short-chain fatty acids measured at two time points (D1 and D9). Strong positive autocorrelations are observed for all metabolites between D1 and D9, with particularly high coefficients for indole-3-propionic acid (IPA) (IPA_D1–IPA_D9), tryptophan (Trp) (Trp_D1–Trp_D9), indole-acetic acid (IAA) (IAA_D1–IAA_D9), and indole-lactic acid (ILA) (ILA_D1–ILA_D9). Within the D1 measurements, pronounced positive correlations are evident between Trp_D1 and IPA_D1, as well as between ILA_D1 and IAA_D1, indicating coordinated variability within the indole metabolic pathway. Among the short-chain fatty acids, notable positive correlations occur between valeric acid (VA) and isovaleric acid (iVA) at D1 (VA_D1–iVA_D1), between butyric acid (BA) and VA (BA_D1–VA_D1), and between caproic acid (CA) and both VA and iVA (CA_D1–VA_D1; CA_D1–iVA_D1). Several SCFA relationships remain consistent at D9, including strong correlations between VA_D9 and iVA_D9, BA_D9 and VA_D9, and CA_D9 with both VA_D9 and iVA_D9 (Figure 4).

To explore the relationship between early metabolite alterations and systemic inflammatory activation, supplementary Spearman correlation analyses were performed. D1 tryptophan levels demonstrated significant moderate negative correlations with admission inflammatory markers (CRP: *ρ* = −0.423, *p* < 0.001; NLR: ρ = −0.421, *p* = 0.001). In contrast, no significant correlations were observed between inflammatory markers and D1 propionic acid (CRP: *ρ* = −0.119, *p* = 0.340; NLR: *ρ* = −0.108, *p* = 0.405).

## 4 Discussion

Our study revealed distinct temporal alterations in gut microbiota–derived metabolites after aSAH, which were strongly associated with both functional outcome and the development of DCI. Patients with unfavorable 3-month outcomes showed lower Day 1 levels of propionic, isobutyric, and isovaleric acids, together with consistently reduced tryptophan concentrations at both time points, while IPA was markedly lower by Day 9. In contrast, favorable outcomes were characterized by higher tryptophan across both sampling points and higher Day 9 IPA levels. DCI was associated with significantly lower tryptophan and propionic acid levels on both Days 1 and 9, as well as a pronounced reduction in IPA on Day 9, and early decreases in isovaleric and caproic acids further distinguished patients who developed DCI. ROC analyses identified tryptophan and propionic acid as the strongest discriminators of both outcome and DCI, with IPA gaining predictive strength at Day 9. Logistic regression demonstrated that lower Day 1 propionic acid and lower Day 9 IPA independently predicted unfavorable functional outcomes, while Day 9 IPA, tryptophan, and propionic acid—as well as Day 1 isovaleric acid and Day 1 caproic acid—were independent predictors of DCI. Correlation analyses showed stable metabolite profiles over time and coordinated clustering within both SCFA and indole metabolic pathways.

Serum tryptophan levels were consistently lower in both the unfavorable outcome and DCI groups at both time points, showed excellent discriminatory accuracy, and Day 9 levels independently predicted DCI after adjustment for clinical covariates. Importantly, the principal associations remained significant after FDR correction, supporting the robustness of the findings.

Our research group has previously demonstrated that patients with poor prognosis after aSAH exhibit significantly lower serum tryptophan levels and higher kynurenine levels (16). Our findings complement prior evidence implicating disruptions in microbiota-derived tryptophan metabolism in neurovascular vulnerability (17) by demonstrating that specific Trp-related and SCFA metabolites independently predict both functional outcome and delayed cerebral ischemia after aSAH, thereby reinforcing a shared pathophysiological axis in which impaired microbial metabolic signaling contributes to early brain injury and its delayed complications. A study by Kofler et al. (18) and our findings highlight tryptophan depletion as a key biological consequence of SAH, but they link it to different clinical manifestations. While Kofler et al. demonstrate that reduced brain tryptophan is associated with depressive symptoms after SAH, our results show that lower systemic tryptophan levels are strongly related to poor functional outcome and the development of DCI. Together, these findings suggest that impaired tryptophan metabolism represents a common pathophysiological axis influencing both neuropsychological vulnerability and neurovascular instability after SAH.

Reduced circulating tryptophan levels in patients with unfavorable outcome after aSAH are likely driven by several converging biological mechanisms. First, the most compelling explanation is inflammation-induced activation of indoleamine-2,3-dioxygenase (IDO1/IDO2) (19), which is rapidly upregulated by cytokines such as IFN-*γ*, IL-6, and TNF-*α* during the acute neuroinflammatory response to hemorrhage. This enzyme diverts tryptophan into the kynurenine pathway, thereby depleting systemic tryptophan stores and generating metabolites associated with vascular dysfunction and secondary brain injury (20). Second, aSAH triggers a profound stress response with activation of the hypothalamic–pituitary–adrenal axis, leading to glucocorticoid-mediated stimulation of hepatic tryptophan-2,3-dioxygenase (TDO) (21–23). Persistent elevations in cortisol in clinically more severe or DCI-prone patients may further accelerate tryptophan catabolism over time, consistent with the sustained reduction observed by Day 9. Third, the metabolic signature of reduced IPA and short-chain fatty acids in our cohort suggests significant gut microbiota dysbiosis, which is known to influence tryptophan availability by altering microbial Trp consumption, impairing indole pathway metabolism, and weakening intestinal barrier integrity (20–24). Such dysbiosis can enhance systemic inflammation and, in turn, amplify IDO activation, creating a self-reinforcing cycle of tryptophan depletion (25). Fourth, alterations in albumin binding and circulating free fatty acids during the acute-phase response may modulate the distribution of free versus bound tryptophan, potentially reducing its bioavailability even when total tryptophan concentrations remain measurable (26). Finally, although nutritional intake is often compromised in critically ill patients, malnutrition alone is unlikely to account for the early and pronounced tryptophan reductions (20, 26), particularly the Day 1 differences observed before sustained changes in feeding status could develop. Together, these mechanisms suggest that tryptophan depletion after aSAH reflects an integrated host response involving inflammation, stress endocrinology, and gut microbial dysregulation—each contributing to a metabolic environment that may predispose to delayed cerebral ischemia and poor neurological recovery.

In our study IPA did not differ between groups at Day 1 but was markedly reduced by Day 9 in patients with unfavorable outcome and DCI. IPA is a gut microbiota–derived tryptophan metabolite produced mainly by gram-positive bacteria, with key roles in immune regulation, antioxidant defense, gut barrier integrity, microbiota balance, and neuroprotection (27). Based on the IPA–gut barrier–immune axis described by Ren et al. (28), our findings suggest that progressive post-aSAH gut microbiome dysbiosis (12, 13) leads to reduced IPA production, which in turn exacerbates gut barrier dysfunction, systemic inflammation, and IDO-mediated diversion of tryptophan toward the kynurenine pathway, thereby increasing neurovascular vulnerability and contributing to DCI and unfavorable outcome. A study by Peng et al. shows that intracerebral hemorrhage is associated with a sustained reduction in gut microbiota–derived IPA, and that IPA supplementation improves neurological recovery and white matter integrity by enhancing microglial myelin debris clearance through inhibition of Stap1, thereby promoting repair (29). This mechanism directly aligns with our findings, as the delayed but marked reduction of IPA in aSAH patients with DCI and poor outcome similarly suggests that loss of microbiota-derived IPA contributes to ongoing neuroinflammation and impaired recovery rather than the immediate acute injury. Furthermore, IPA directly alleviates ischemic brain injury via anti-inflammatory, antioxidant, and neuroprotective mechanisms (30), our results suggest that the reduced IPA levels observed in aSAH patients with DCI and poor outcome likely reflect loss of an endogenous protective metabolite that normally mitigates secondary ischemic injury and supports neurological recovery. Broad-spectrum antibiotic treatment decreases the relative abundance of *C. difficile* and almost completely inhibits IPA synthesis, reducing levels to nearly zero (31); however, although infections and consequent antibiotic use were significantly more frequent in the unfavorable aSAH group in our study and may therefore influence IPA levels, our logistic regression analysis demonstrated that low IPA remained an independent predictor of outcome. Taken together, aSAH-associated gut dysbiosis likely reduces microbial IPA synthesis from tryptophan, while the consequent loss of IPA-mediated barrier-protective and immunomodulatory signaling promotes gut permeability and systemic inflammation, diminishing IPA’s endogenous neuroprotective effects against delayed ischemic injury and DCI and further reinforcing IPA depletion through inflammation-driven IDO activation and diversion of tryptophan away from indole metabolite production. Across analyses in our study, lower levels of specific SCFAs were consistently associated with worse outcomes, as propionic, isobutyric, isovaleric, and caproic acids were reduced in patients with unfavorable functional outcome and/or DCI—particularly early after admission—with propionic acid showing the strongest and most consistent independent predictive value for both poor outcome and DCI.

Accumulating evidence across ischemic stroke, traumatic brain injury (TBI), intracerebral hemorrhage (ICH), and aneurysmal subarachnoid hemorrhage (aSAH) supports a central role for gut microbiota–derived short-chain fatty acids (SCFAs) in shaping post-injury inflammation, secondary brain damage, and neurological recovery. Experimental stroke studies demonstrate that reductions in SCFA availability impair functional recovery, whereas restoration of SCFAs improves outcome by modulating adaptive immune responses, microglial activation, and neuroplasticity (32, 33). Human studies extend these findings by showing that acute brain injury is accompanied by gut dysbiosis and altered circulating SCFA levels that associate with functional outcome and inflammatory markers, although the direction and strength of these associations depend on the timing of measurement and clinical context (7, 34).

In this context, our observation that propionic acid is already reduced at Day 1 in patients with unfavorable outcome and independently predicts DCI at Day 9 suggests that early propionate depletion reflects a loss of SCFA-mediated immunoregulatory control during the hyperacute phase after aSAH. This interpretation is consistent with ischemic stroke data linking lower SCFA levels to poorer recovery (7) and with aSAH studies showing that gut microbiome profiles with altered metabolic capacity are associated with vasospasm and DCI, implying that an early pro-inflammatory metabolic milieu may predispose patients to secondary ischemic injury (12, 35).

The temporal pattern observed for isobutyric acid further underscores the dynamic nature of SCFA signaling after brain injury. Although isobutyric acid is consistently lower in patients with unfavorable outcome, its independent association emerges only at Day 9, suggesting that later SCFA measurements may better capture persistent gut dysbiosis and ongoing neuroinflammation rather than acute injury severity. This aligns with thrombectomy-based stroke studies in which acute SCFA levels correlated more strongly with systemic inflammatory markers than with initial neurological deficit, while longer-term associations appeared more relevant for recovery (34), and with ICH studies demonstrating that sustained reductions in SCFA-producing taxa are linked to impaired neurological improvement (36, 37).

Notably, the early reductions in isovaleric and caproic acids observed in patients who subsequently developed DCI point toward a role for branched-chain and medium-chain SCFAs as early metabolic signals of vulnerability to secondary injury. While individual SCFAs have been less extensively studied in hemorrhagic stroke, multi-omics analyses in ICH indicate that microbiome-driven alterations in host metabolism, including SCFA-related pathways, closely track disease severity and outcome (38). Within this framework, early deficits in isovaleric and caproic acids may reflect acute disruption of the gut–brain axis that precedes and contributes to delayed ischemic complications after aSAH (12, 35). Moreover, evidence from traumatic brain injury models demonstrates that sustained post-injury gut dysbiosis and reduced SCFA availability are associated with worse long-term neurological outcomes, whereas restoration of SCFA signaling—either through microbial recovery or direct supplementation—attenuates neuroinflammation and improves functional recovery, further supporting a causal role of SCFA deficiency in secondary brain injury across acute brain insults (39–41). Mechanistic insights from experimental stroke and TBI models provide biological plausibility for these clinical observations by demonstrating that SCFAs directly regulate immune cell differentiation, T-cell trafficking, microglial activation, and blood–brain barrier integrity, thereby influencing both secondary injury cascades and long-term recovery trajectories (32, 33, 42). At the same time, human studies emphasize that circulating SCFA levels in the acute phase may represent a composite signal integrating compensatory responses, metabolic stress, and treatment-related factors, which may explain time-dependent or even paradoxical associations with outcome (34). In supplementary analyses, early tryptophan levels demonstrated significant inverse correlations with admission inflammatory markers (CRP and NLR), supporting an inflammation-associated metabolic shift consistent with activation of the indoleamine 2,3-dioxygenase (IDO) pathway. In contrast, early propionic acid levels did not correlate with inflammatory markers, suggesting that SCFA alterations may reflect distinct regulatory mechanisms beyond acute systemic inflammatory burden. Importantly, although early reductions in tryptophan and propionic acid were independently associated with DCI and unfavorable outcome, the observational nature of this study precludes causal inference. Reduced metabolite levels may represent biologically active contributors to secondary brain injury or may function as markers of systemic inflammation and overall disease severity. Therefore, these findings should be interpreted as associative rather than mechanistic evidence of causality. Further experimental and interventional studies will be necessary to clarify the directionality and biological relevance of these associations.

Several limitations of the present study should be acknowledged. First, this was a single-center, observational study with a relatively modest sample size, which may limit the generalizability of our findings. Although the cohort size is comparable to or larger than many metabolomic studies in aSAH, external validation in independent, multicenter cohorts is required before these biomarkers can be reliably applied in broader clinical settings. Antibiotic exposure represents an important potential confounder in studies investigating gut microbiota–derived metabolites. In the present cohort, no patient received antibiotics prior to D1 sampling and no routine prophylaxis was applied; thus, early (D1) metabolite alterations cannot be attributed to antibiotic treatment. However, most antibiotic therapies were initiated before or during the DCI risk window (median initiation 7 days after D1), which may have influenced D9 metabolite levels—particularly IPA—through microbiome disruption. Although infection status was included as a covariate in adjusted outcome models, residual confounding by antibiotic type, duration, and timing cannot be entirely excluded. Future studies with detailed longitudinal antibiotic exposure data are warranted to further clarify this effect. Second, the observational design precludes causal inference. While strong associations were identified between specific gut microbiota–derived metabolites, functional outcome, and delayed cerebral ischemia (DCI), our data cannot establish whether altered metabolite levels are mechanistic contributors to secondary brain injury or epiphenomena reflecting disease severity, systemic inflammation, or treatment-related factors. Third, metabolite measurements were limited to two predefined time points (Day 1 and Day 9). Although these time points were selected to capture the acute phase and the peak risk window for DCI, they may not fully reflect the dynamic and potentially nonlinear temporal trajectories of microbiota-derived metabolites after aSAH. More frequent longitudinal sampling could provide deeper insight into the evolution of gut–brain metabolic interactions. Fourth, direct assessment of gut microbiota composition was not performed. Consequently, the observed alterations in circulating SCFAs and indole metabolites can only indirectly reflect gut dysbiosis. Integration of fecal microbiome sequencing with serum metabolomics would be necessary to directly link microbial taxa, metabolic capacity, and clinical outcomes. Fifth, several potential confounders inherent to neurocritical care may have influenced metabolite levels, including antibiotic exposure, enteral nutrition practices, mechanical ventilation, infections, and other intensive care interventions. Although infection status was included as a covariate in multivariable models, residual confounding cannot be excluded. Finally, nutritional intake and metabolic status prior to hemorrhage were not systematically quantified, and baseline pre-aSAH metabolite levels were unavailable. Therefore, interindividual variability in premorbid metabolism may have contributed to the observed differences. Additionally, the absence of a formal *a priori* sample size calculation may limit statistical power for detecting smaller effect sizes. Because the median time to DCI was 9 days, approximately half of the DCI events occurred before or on the Day 9 sampling time point. Therefore, Day 9 metabolite levels should be interpreted as reflecting dynamic pathophysiological processes during the peak DCI window rather than as purely prospective predictors. Despite these limitations, the consistency of findings across multiple analytical approaches and time points supports the robustness of the observed associations and highlights the need for future mechanistic and interventional studies.

## 5 Conclusion

In conclusion, our present findings integrate well with the existing literature and support a model in which early depletion of specific SCFAs—particularly propionic, isovaleric, and caproic acids—marks an increased risk for DCI, whereas persistently low SCFA levels at later time points, such as isobutyric acid at Day 9, reflect sustained dysbiosis and impaired recovery potential. This temporal and metabolite-specific pattern reinforces the concept that SCFAs are not merely bystanders but dynamic indicators of gut–brain–immune interactions after aSAH, with potential implications for risk stratification and targeted therapeutic interventions.

## Data Availability

The original contributions presented in the study are included in the article/Supplementary material, further inquiries can be directed to the corresponding author.
