# Supplementary material for: Serum microbiome-related metabolites—including short-chain fatty acids and indole derivatives—predict outcome and delayed cerebral ischemia after aneurysmal subarachnoid hemorrhage: a two-timepoint LC–MS study
Source: Front Neurol. 2026 Apr 7;17:1768108. doi: 10.3389/fneur.2026.1768108 (PMC13095518; doi:10.3389/fneur.2026.1768108)
Supplement: Supplementary file 1 [file Image_1.pdf]

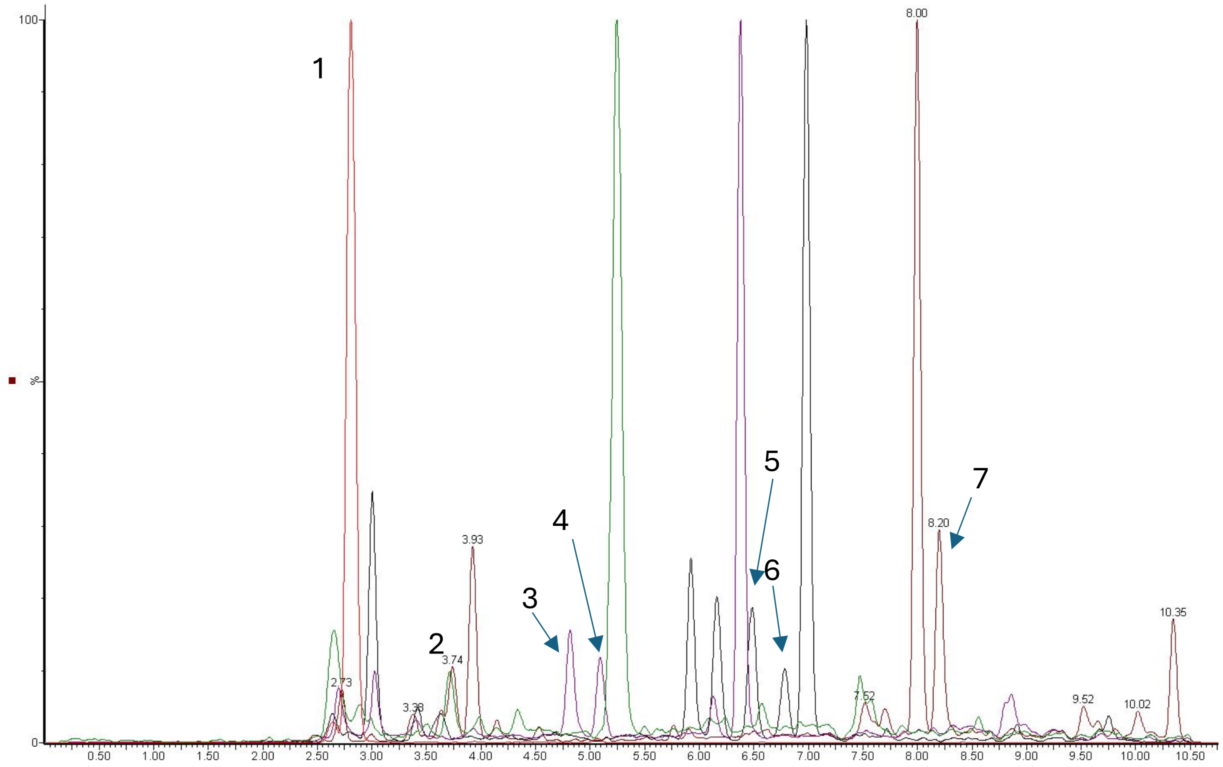

Supplementary Figure S1. Representative MRM chromatogram of SCFA compounds obtained from pooled serum extract.

The chromatogram demonstrates adequate chromatographic separation and peak specificity for all quantified SCFAs following derivatization. Peaks correspond to: (1) acetic acid, (2) propionic acid, (3) butyric acid, (4) isobutyric acid, (5) isovaleric acid, (6) valeric acid, and (7) caproic acid.
