# Supplementary material for: Serum microbiome-related metabolites—including short-chain fatty acids and indole derivatives—predict outcome and delayed cerebral ischemia after aneurysmal subarachnoid hemorrhage: a two-timepoint LC–MS study
Source: Front Neurol. 2026 Apr 7;17:1768108. doi: 10.3389/fneur.2026.1768108 (PMC13095518; doi:10.3389/fneur.2026.1768108)
Supplement: Supplementary file 2 [file Table_1.DOCX]

|  | technical CV calculated from the multiple injection of single derivatized pooled extract (%) | derivatization CV across multiple batches (%) | S/N in the average of blank samples | S/N in lowest point of the calibration | The concentration of lowest point of calibration is considered as LOQ ng/ml |
| --- | --- | --- | --- | --- | --- |
| Propionic acid | 2.2 | 31.2 | 125.000 | 290.000 | 9.259 |
| Butyric acid | 1.5 | 20.2 | 19.333 | 43.000 | 9.259 |
| Isobutyric acid | 2.2 | 15.6 | 6.250 | 13.000 | 9.259 |
| Valerianic acid | 2.1 | 19.4 | 10.333 | 26.500 | 4.630 |
| Isovalerianic acid | 3.5 | 12.3 | 2.500 | 17.500 | 4.630 |
| Caproic acid | 3.1 | 21.4 | 50.500 | 117.000 | 4.630 |

Supplementary Table S1. Analytical performance parameters of SCFA quantification
