# Supplementary material for: Serum microbiome-related metabolites—including short-chain fatty acids and indole derivatives—predict outcome and delayed cerebral ischemia after aneurysmal subarachnoid hemorrhage: a two-timepoint LC–MS study
Source: Front Neurol. 2026 Apr 7;17:1768108. doi: 10.3389/fneur.2026.1768108 (PMC13095518; doi:10.3389/fneur.2026.1768108)
Supplement: Supplementary file 3 [file Table_2.DOCX]

|  | calibration curve | R^2^ | linear range ng/ml |
| --- | --- | --- | --- |
| Propionic acid | 79.61*X + 3341 | 0.998 | 148.148 - 1333 |
| Butyric acid | 459.787*X + 4374 | 0.999 | 37.037 - 1333 |
| Isobutyric acid | 134.554*X + 925 | 0.999 | 37.037 - 1333 |
| Valerianic acid | 302.768*X + 1330 | 0.997 | 18.519 - 666.667 |
| Isovalerianic acid | 341.233*X + 429 | 0.998 | 9.259 - 666.667 |
| Caproic acid | 69.49*X + 715.2 | 0.999 | 74.074 - 666.667 |

Supplementary Table S2. Calibration parameters for SCFA quantification
